# Bronchoscopic lung volume reduction by instillation of fibrinogen and thrombin in COPD patients with homogenous emphysema

**DOI:** 10.1186/s12890-024-02883-4

**Published:** 2024-02-14

**Authors:** Dina Abd El Wahab El Gohary, Mohamed Adel Eltomey, Ahmed Shawky Mohamed, Amgad Abd El Raouf Farahat, Ayman Hassan Abd El Zaher

**Affiliations:** 1 Chest Diseases Department, Faculty of Medicine, Tanta University, Tanta, Egypt; 2 Diagnostic Radiology Department, Faculty of Medicine, Tanta University, Tanta, Egypt

**Keywords:** Fibrinogen and thrombin, Biologic lung volume reduction, Homogenous emphysema

## Abstract

**Background:**

The new endobronchial therapy called biological lung volume reduction (BioLVR) involves using a rapid polymerizing sealant to block off the most emphysematous portions of the lungs. The primary mechanism of action is resorption atelectasis, which is then followed by inflammation and remodeling of the airspace. The remodeling process will result in the formation of scars, leading to the contraction of the lung tissue. As a result, a decrease in functional lung volume is anticipated for a period of 6–8 weeks.

**Objective:**

Assessing the safety and effectiveness of bronchoscopic installation of (fibrinogen and thrombin) in COPD patients with homogeneous emphysema in terms of radiological, physiological, and quality of life outcomes.

**Methods:**

Between December 2017 and December 2019, 40 COPD patients with homogeneous emphysema were studied using a fiber optic bronchoscope while they were awake but sedated. Tanta University Hospitals’ chest medicine department collaborated with the diagnostic radiology department of the Faculty of Medicine.

**Results:**

All the following parameters were reduced from their initial values: HRCT volumetry, RV/TLC, mMRC dyspnea scale, CAT score, 6MWT, FEV1, and the FEV1/FVC ratio at the first, third, and sixth months from the beginning (*p* = 0.001). One individual (0.025%) had pneumonia, whereas three individuals had COPD (0.075%). Using fibrin glue produced locally, biological lung volume reduction (Bio LVR) may be an effective treatment for advanced homogenous emphysema.

**Conclusion:**

By using locally prepared fibrin glue the biologic lung volume reduction (Bio LVR) may be a convenient method to treat advanced homogenous emphysema.

## Introduction

(COPD) is a preventable, prevalent, and curable lung disease, which is characterized by abnormalities at the airway and/or the alveoli due to frequent exposure to noxious particles or gases [1]. **(**BioLVR) is a novel endobronchial technique that involves occluding the most emphysematous areas of the lung with a sealant that polymers quickly. Within 6 to 8 weeks, airway occlusion causes resorption atelectasis, inflammation and scarring in the airspace, constriction of the lung parenchyma, and a decrease in functional volume [2, 3]. 

## Patients and methods

This research plan was accepted by the Tanta University Ethical Committee with approval number 31,824/10/17. Informed consent was given for the procedure by the patients or their families. This prospective interventional cohort study was conducted from December 2017 to February 2019 with 40 COPD patients who had homogenous emphysema. The study was conducted in cooperation with Tanta University Hospitals’ diagnostic radiology department.

### Study subjects

According to the GOLD stage of severity of airflow limitation, all trial participants had advanced homogenous emphysema, moderate to severe airflow limitation, respiratory complaints in spite of receiving appropriate medical treatment, and were either refused surgery for lung volume reduction or were ineligible for it.

### Inclusion standards

- The patient’s age is forty years or more.
- A high resolution computed tomography (HRCT) indicated the presence of homogenous emphysema.
- According to the most current Medical Research Council, patients have grade 2 dyspnea (mMRC) or more.
- The post-bronchodilator (forced expiratory volume in one second to forced vital capacity ratio) (FEV1/FVC) was 70% or less, and the FEV1 ranged from (30–50%) of the predicted value.
- Total lung capacity (TLC) of 75% or more of the expected value and residual volume (RV) of 150% or more suggest hyperinflation.
- A former smoker who smoked for 20 pack-years.
- Patients who have declined lung volume reduction surgery or who do not qualify for the procedure.
- Patients who might potentially benefit from a bronchoscopy.

### Exclusion standards

- Reduction in lung capacity or prior lobectomy; clinically severe bronchitis or asthma.
- The most recent severe chronic obstructive pulmonary disease (COPD) flare-up occurred less than 2 months prior to the procedure.
- Imaging findings that are consistent with a lung nodule bigger than 1 cm in diameter, a huge bullous disease (predominant bulla larger than 10 cm in diameter), significant interstitial lung disease, or pleural disease.
- HIV, serious malignancy, and recent myocardial infarction are examples.
- The incidence of pulmonary hypertension is prevalent and estimated by echocardiography (estimated systolic pulmonary artery pressure > 45 mmHg).
- The BMI ranges between 15 and 35 kg/m2.

### The following procedures were applied to all chosen patients

Complete blood count (CBC), partial thromboplastin time (PTT), prothrombin time (PT), international normalized ratio (INR), electrocardiogram (ECG), arterial blood gases (ABG), kidney and liver function tests are performed after a thorough medical history and physical examination have been obtained.

- Baseline values were obtained from the following assessments on admission and at 1-, 3-, and 6-months post-op:
- Chest CT with many detectors (HRCT volumetry).
- Using single-breath helium dilution technology, European manufacturers of medical equipment provide very accurate pulmonary function tests (spirometry; FEV1, FVC, FEV1/FVC lung volumes; RV, TLC, and RV/TLC).
- 6 min walk distance (6MWD).
- The dyspnea rating scale was developed by the mMRC.
- Quality of life is evaluated with the use of the COPD Assessment Test (CAT) score.
- High-Resolution Computed Tomography of Large Volumes (HRCT).
- HRCT volumetry was performed using a 128-detector CT scanner (a Philips Ingenuity Core128 scanner from Philips Medical Systems in Best, the Netherlands, which is also capable of helical CT).

**Fig. 1:**
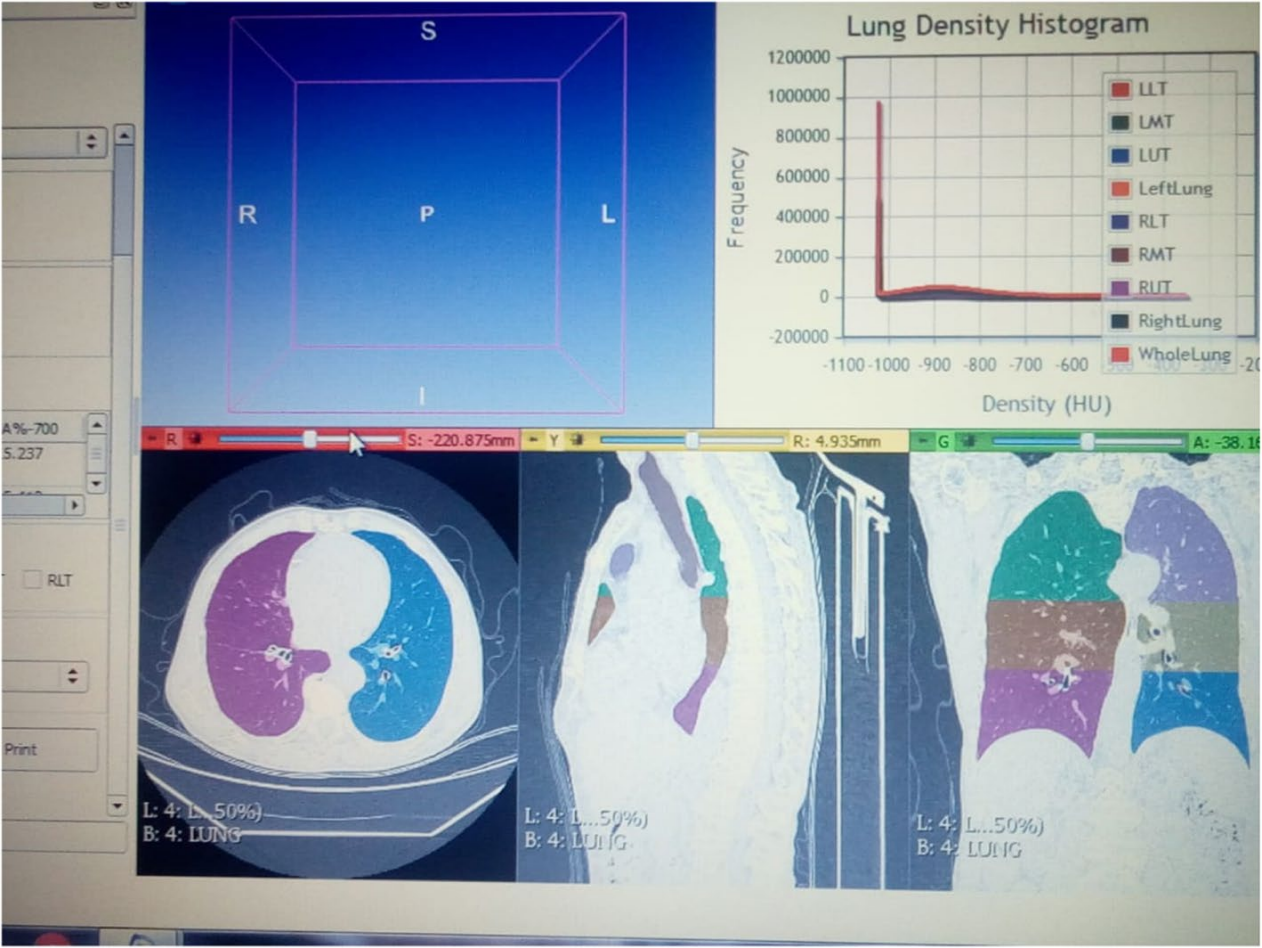
Slicer chest imaging plateform

Digital Imaging and Communications in Medicine (CT DICOM) (pictures of homogenous emphysema were evaluated by radiologists in the Diagnostic Radiology Department. The radiology team received the pictures and analyzed them using commercial software (Pulmonary Workflow Plus) to validate the homogenous phenotype, showing that the ratio of upper to lower lobe voxels at -950 HU was between 0.98 and 1.02 (i.e., 1.0 0.02) [4] (Fig. 1).

After administering 2–5 mg of midazolam and 3–5 puffs of 10% lidocaine spray as a local anesthetic, a fiberoptic bronchoscope (PENTAX LH-150PC) was used to perform a bronchoscopy on the patient.

The patient’s blood oxygen level was monitored as more oxygen was administered via a nasal catheter when needed.

The bronchoscope was inserted into the patient’s airway by the mouth or nose and guided to the segmental bronchus identified by HRCT of the chest.

Fibrin glue was made locally by mixing fibrinogen with thrombin-containing plasma. Blood samples containing fibrinogen were previously prepared by adding citrate to the blood, then centrifuging the mixture to separate the plasma from the rest of the blood. This was a sterile operation performed by the Clinical Pathology Department clean, germ-free atmosphere.

The SIEMENS Multifibrin-U, a commercially available reagent, was used to produce thrombin (Siemens Healthcare Diagnostics Products Gmbh, Marburg, Germany) [5]. 

The Clinical Pathology Department made many efforts to determine the plasma-to-thrombin concentration range that would result in a coagulum.

**Fig. 2:**
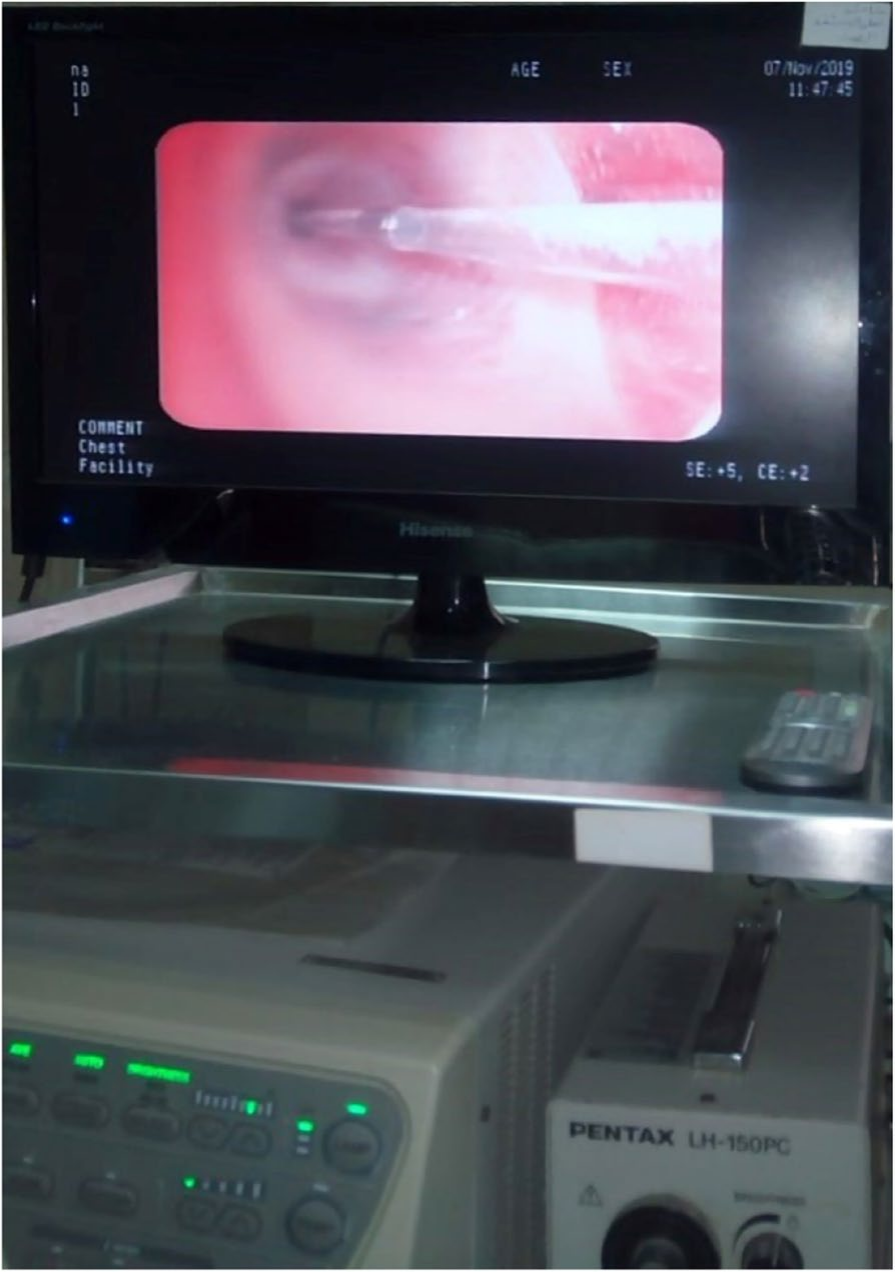
Triple lumen balloon catheter inserted into the targeted site

 For this study, 10 mL of fibrin glue were made from 5 mL of plasma and 5 mL of thrombin. Everything was glued together using this glue. After the balloon was inflated, the patient’s plasma and thrombin were given simultaneously via the Triple lumen catheter to the treatment site. Fibrin glue was allowed to develop in the target location by leaving the catheter in place for two minutes. For the succeeding segment of focused analysis, the method was employed once again in a separate session (Fig. 2).

### Post maneuver follow-up for early and late complications

Screening the (COPD) patients for immediate and long-term complications as pneumothorax, heart ischemia, pulmonary infarction, and 24 h after surgery as lung abscess and empyema.

### Outcomes evaluation

#### Primary outcome

One month after maneuver, a three-dimensional volumetric HRCT was utilized to quantify the selected area of emphysema and a reduction in (RV/TLC percent anticipated) from baseline.

#### Measures to minimize the risks

Patients were closely observed, and any problems that occurred were dealt with skillfully. There was extreme hygiene in the way the surgery was carried out. Three days before to the therapy, antibiotics were given, and painkillers were then given for a week.

#### Secondary outcome

Changes in mMRC, CAT, 6MWD, FEV1% predicted after bronchodilator, and FEV1/FVC ratio after 3, 6, and 12 months compared to baseline.

### Statistical analysis

To interpret the data, the Statistical Program for Social Science, version 24.0, was utilized. Quantitative data analysis using the mean and standard deviation (SD). used qualitative data, including quantity and frequency. When comparing the means of more than two groups, a one-way analysis of variance (ANOVA) is performed. Correlations between data sets have been established using Pearson’s correlation coefficient (r) test.

## Results

 In terms of patient demographics, forty male patients with range of age from 44 to 71 years old were included in this study, with a mean age (58.40 ± 6.86 years) with a variety of smoking indexes (20–100) pack/year and mean of smoking index is (51.35 ± 22.56) (Table 1).

**Table 1:** Patient demographics

Variable	Range	Mean ± SD
Age	44–71	58.40 ± 6.86
Smoking index	20–100	51.35 ± 22.56

There were 15 COPD patients (37.5%) with right upper lobar emphysema, 13 patients (32.5%) with left upper lobe emphysema, 7 patients (17.5%) with left lower lobe emphysema, and 5 patients (12.5%) with right lower lobe emphysema (Table 2).

**Table 2:** Distributions of emphysema in COPD patients

Emphysema distribution (Patients No.= 40)	No	%
Right upper lobe	15	37.5%
Right lower lobe	5	12.5%
Left upper lobe	13	32.5%
Left lower lobe	7	17.5%

Table 3 showed that compared to pre-BLVR levels, mMRC dyspnea scores dropped significantly at 1, 3, and 6 months after BLVR.

**Table 3:** Comparison of the mMRC dyspnea scale before and after BLVR at the 1st, 3rd, and 6th month

mMRC	Baseline	1 month		3 months	6 months
Mean ± SD	3.35 ± 0.53	2.43 ± 0.50		2.40 ± 0.50	2.38 ± 0.49
F test	35.395				
*P* value	0.001*				
Baseline & 1 m.	Baseline & 3 m.	Baseline & 6 m.	1 m. & 3 m.	1 m. & 6 m.	3 m. & 6 m.
0.001*	0.001*	0.001*	0.996	0.971	0.996

* : *p* value < 0.05

Statistical analysis of pre- and post-BLVR PaO2 at 1, 3, and 6 months showed a substantial increase in PaO2 at all three time points (Table 4).

**Table 4:** Comparison between PaO_2_ before BLVR and at and 1^st^, 3^rd^ and 6^th^ month post procedure

PaO_2_/mmHg	Baseline	1 month		3 months	6 months
Mean ± SD	61.03 ± 3.42	71.88 ± 7.33		73.15 ± 7.06	73.73 ± 6.89
F test	35.348				
*P* value	0.001*				
Baseline & 1 m.	Baseline & 3 m.	Baseline & 6 m.	1 m. & 3 m.	1 m. & 6 m.	3 m. & 6 m.
0.001*	0.001*	0.001*	0.808	0.566	0.978

* : *p* value < 0.05

Table 5 showed that there was a statistically significant increase in FEV1/FVC (percent) at the 1^st^, 3^rd^, and sixth months following BLVR as compared to FEV1/FVC (percent) before BLVR.

**Table 5:** Comparison between FEV1/FVC (%) before BLVR and at 1^st^, 3^rd^ and 6^th^ month post procedure

FEV1/FVC (%)	Baseline	1 month		3 months	6 months
Mean ± SD	39.30 ± 8.55	49.58 ± 9.49		52.75 ± 9.13	54.35 ± 9.13
F test	22.171				
*P* value	0.001*				
Baseline & 1 m.	Baseline & 3 m.	Baseline & 6 m.	1 m. & 3 m.	1 m. & 6 m.	3 m. & 6 m.
0.001*	0.001*	0.001*	0.402	0.091	0.860

* : *p* value < 0.05

Table 6 showed that there was statistically significant improvement in FEV1% at 1^st^, 3^rd^ and 6^th^ month post procedure.

**Table 6:** Comparison between FEV1 (%) of predicted before BLVR and at 1^st^, 3^rd^ and 6^th^ month post procedure

FEV1 (%)	Baseline	1st month		3rd months	6th months
**Mean ± SD**	32.73 ± 4.29	41.03 ± 6.49		43.65 ± 6.47	44.28 ± 6.34
**F test**	76.363				
***P*** **value**	0.001*				
**Baseline & 1 m.**	**Baseline & 3 m.**	**Baseline & 6 m.**	**1 m. & 3 m.**	**1 m. & 6 m.**	**3 m. & 6 m.**
0.001*	0.001*	0.001*	0.205	0.075	0.966

* : *p* value < 0.05

 Regarding (RV/TLC (percent)), there was statistically significant reduction (improvement) when comparing the baseline before BLVR with post treatment at the 1^st^, 3rd, and sixth months (Table 7).

**Table 7:** Comparison between RV/TLC (%) before BLVR and at 1^st^, 3^rd^ and 6^th^ month post procedure

RV/TLC (%)	Baseline	1 month		3 months	6 months
Mean ± SD	166.28 ± 11.87	126.55 ± 17.28		115.85 ± 17.68	107.20 ± 18.94
F test	98.119				
*P* value	0.001*				
Baseline & 1 m.	Baseline & 3 m.	Baseline & 6 m.	1 m. & 3 m.	1 m. & 6 m.	3 m. & 6 m.
0.001*	0.001*	0.001*	0.024*	0.001*	0.098

* : *p* value < 0.05

**Fig. 3:**
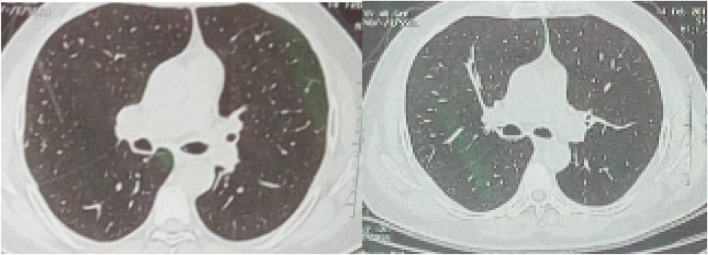
Base line and follow up HRCT of patient in whom BLVR was done with fibrin glue at 1^st^ month post procedure

**Fig. 4:**
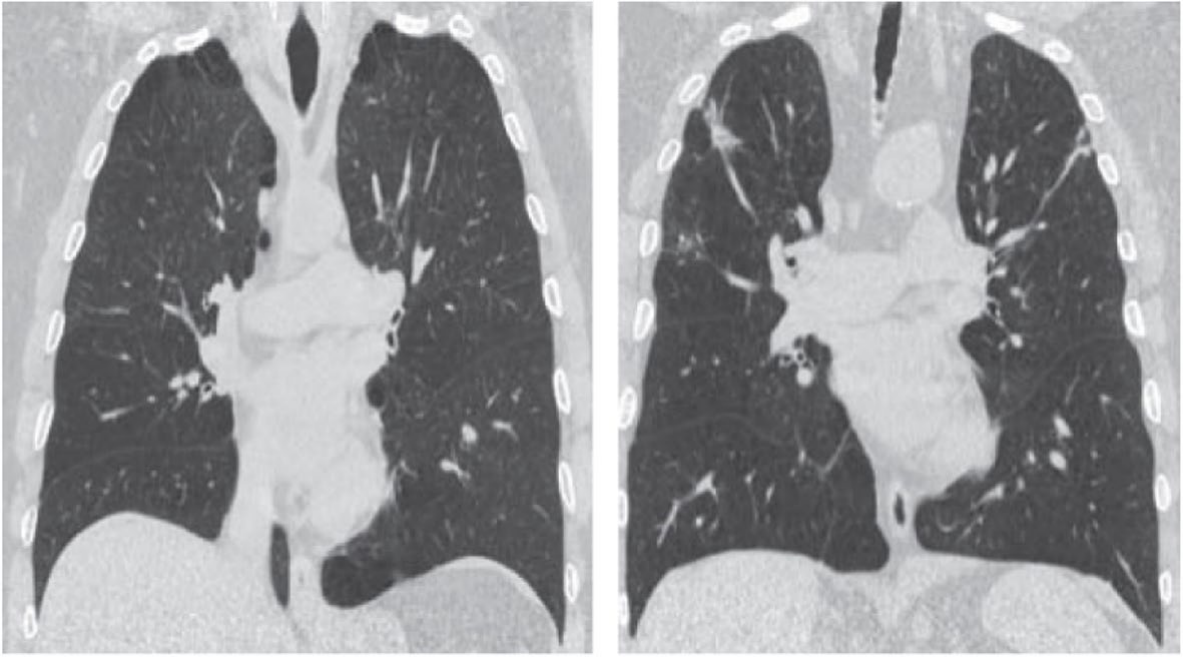
Baseline and 3^rd^ month post BLVR (CT) changes. Coronal CT imaging in a patient in each upper lobe. Scarring effect were observed

 In the first, third, and sixth months after treatment, lung volumetry decreased significantly due to scarring responses (Table 8) and (Figs. 3 and 4).

**Table 8:** Comparison between HRCT volumetry before BLVR and at 1^st^, 3^rd^ and 6^th^ month post procedure

HRCT volumetry (ml^3^)	Baseline	1 month		3 months	6 months
Mean ± SD	1398.60 ± 585.08	1037.50 ± 503.25		926.03 ± 451.54	860.91 ± 430.50
F test	9.285				
*P* value	0.001*				
Baseline & 1 m.	Baseline & 3 m.	Baseline & 6 m.	1 m. & 3 m.	1 m. & 6 m.	3 m. & 6 m.
0.008*	0.001*	0.001*	0.747	0.393	0.937

* : *p* value < 0.05

 Results from a comparison of 6MWD before BLVR with 6MWD after the first, third, and sixth months of treatment are given in (Table 9). All three time points demonstrate a statistically significant improvement in 6MWD.

**Table 9:** Comparison between 6MWD before BLVR and at 1^st^, 3^rd^ and 6^th^ month post procedure

6MWD	Baseline	1 month		3 months	6 months
Mean ± SD	404.58 ± 73.39	453.28 ± 76.87		466.53 ± 73.77	482.65 ± 77.21
F test	7.991				
*P* value	0.001*				
Baseline & 1 m.	Baseline & 3 m.	Baseline & 6 m.	1 m. & 3 m.	1 m. & 6 m.	3 m. & 6 m.
0.023*	0.002*	0.001*	0.860	0.305	0.774

* : *p* value < 0.05

 When patients’ CAT scores were evaluated before and after BLVR, as well as after 3 and 6 months of recovery, they showed a significant decrease (Table 10).

**Table 10:** Comparison between CAT score before BLVR and at 1^st^, 3^rd^ and 6^th^ month post procedure

CAT	Baseline	1 month		3 months	6 months
Mean ± SD	34.68 ± 3.58	29.58 ± 2.60		28.65 ± 1.94	27.35 ± 1.55
F test	64.024				
*P* value	0.001*				
Baseline & 1 m.	Baseline & 3 m.	Baseline & 6 m.	1 m. & 3 m.	1 m. & 6 m.	3 m. & 6 m.
0.001*	0.001*	0.001*	0.364	0.001*	0.104

* : *p* value < 0.05

 Three patients (7.5%) had an exacerbation of their COPD, whereas only one (2.5%) patient who underwent maneuver developed pneumonia (Table 11).

**Table 11:** Post procedure complications

Complication	No (40)	%
COPD excerbation	3	7.5
Pneumonia	1	2.5
Haemoptysis	0	0
Pleuritic chest pain	0	0

## Discussion

### Primary outcome

In this study, it was found that there was a reduction in lung volume at 1st,3rd and 6th month post procedure determined by HRCT volumetry and RV/TLC (percent predicted).

In agreement of our study, a study conducted by Bakeer et al. [3], who found reduction in HRCT volumetry at the conclusion of the trial in both group A and group B (*P* = 0.001 and *P* = 0.005, respectively) after examining 15 participants, eight of whom used fibrin adhesive and seven of whom utilized their own blood.

Also Mizumori et al. [6] agreed with our findings regarding reduction in HRCT lung volumetry as they employed trans bronchial injection of autologous blood and thrombin and in the follow up observed a reduction in the emphysematous region confirmed by HRCT lung volumetry.

Refaely et al. [7], in their investigation of 25 patients diagnosed with homogenous emphysema who underwent biological lung volume reduction therapy, observed a radiological response manifested as peripheral atelectasis and scarring reactions. Furthermore, the extent of these reactions was greater when 20 ml per site treatment was implemented as opposed to 10 ml.

Regarding (RV/TLC), it was significantly improved at months 1, 3, and 6 compared to the beginning of treatment (p 0.001).

Reilly et al. [2] found the same results when employed fibrin glue on six patients with advanced heterogeneous emphysema.

the patients were subdivided into two sub groups. the first group received BioLVR on two sub segments and the second group was treated in four sub segments. Efficacy data demonstrated significant decline in RV/ TLC that was more marked in second group.

Criner et al. [8] had found that both high (20 mL/subsegment) and low (10 mL/subsegment) fibrin hydrogel dosage regimens were shown to significantly reduce the RV/TLC ratio after 3 months (*P* = 0.028 and *P* = 0.002, respectively.

Also, (Refaely et al.) [7] showed a significant reduction of hyperinflation in patients treated by BioLVR using 20 mL hydrogel foam per treatment site at eight sub segments.

Consistent with the results of Criner et al. [8], who examined 50 patients with advanced upper lobe emphysema to determine the therapeutic dosage and safety of fibrin hydrogel and observed a significant radiological response at 6 weeks, including scarring, atelectasis, and higher linear densities, our findings indicate a higher incidence of emphysema in the right upper lobe.

### Secondary outcome

There was marvelous improvement regarding; dyspnea (measured by mMRC dyspnea scale), quality of life (measured by CAT score) and exercise capacity (measured by 6MWD) in this study at follow up at 1st, 3rd and 6th month post maneuver.

 Criner et al. [8] agreed with improvement regarding dyspnea as they found that there was improvement of mMRC dyspnea scale in the high dose group (20 mL per site treatment) at 12 weeks follow-up (*P* = 0.002).

 Kramer et al. [9] also noticed significant improvement of mMRC dyspnea scale 12 weeks follow-up (*P* = 0.011), Bakeer et al. [3] had the same results regarding improvement in dyspnea as they studied 50 male patients with heterogeneous emphysema, studied patients were divided into two groups: group A, which included seven patients in whom autologous blood was used and group B, which included eight patients in whom locally prepared fibrin glue was used.

In speaking about 6 min walk distance (6MWD), the results of our study were in agreement with all of the followings; Kobayashi and Kanoh [10] who did BLVR with blood in four patients and found that the 6MWD had improved in two patients, Herth et al. [11] who found increase in a 6MWD after bronchoscopic instillation of hydrogel into the target lobe, also Bakeer et al. [3] found significant increase in 6MWD and Elhanafy et al. [12] found a statistically significant increase in 6 MWD after BLVR.

On the contrary, these findings were in opposition to those of Kramer et al. [9] as there was no significant improvement in 6MWD in the follow-up time (*P* = 0.305) and Criner et al. [8] as there was no significant improvement in 6MWD in the same follow-up time (*P* = 0.705).

There was improvement regarding oxygen saturation in follow–up measurments (*p* value = 0.001) in our study.

This results agreed with Venuta et al. [13] who showed efficient rise of PaO2 after BLVR, also with Elhanafy et al. [12].

 Reilly et al. [2] disagreed with that result as they found that there was no significant change in arterial blood gas value.

Spirometric measures outcomes in this study showed that there was significant change between base line and follow up ( improvement ) regarding FEV1/ FVC and FEV1% (*p* value = 0.001) and (*p* value = 0.001) respectively.

In parallel to these findings; Refaely [7], Criner [8], Bakeer [3] and Atta et al. [14] found a significant improvement in FEV1% after BLVR (*P* value < 0.001) and Elhanafy et al. [12] found significant increase in both FEV1 and FVC, but Reilly et al. [2] found no important changes were detected in FEV1 post maneuver.

In order to determine whether or not the procedure is risk-free, the incidence of severe medical complications linked to bronchoscopic lung volume reduction therapy is evaluated. Neither significant complications occurred during nor after the procedure in this study. In this study, only one patient had pneumonia at the same side of the procedure at the 7th day of maneuver, which improved after 10 days of inpatient treatment, in contrast Kramer et al. [9] reported one patient that developed tension pneumothorax who mechanically ventilated.

Also venuta et al. [13] observed complications in the form of a contralateral pneumothorax that was developed 15 days post procedure.

 Criner et al. [8] reported that only one patient developed aspiration pneumonia after 8 h post maneuver and followed by myocardial infarction.

 Hopkinson et al. [15] found a radiological evidence of ipsilateral pneumothoraces in two patients after BLVR.

In our research, three patients experienced a COPD exacerbation 3 days after the procedure, which was successfully managed with medical intervention. However, Criner et al. [8] documented that nine out of twenty-two high dose patients experienced COPD exacerbations within the initial six months of treatment.

 Refaely et al. [7] reported that COPD exacerbations were noticed in three of siventeen patients in the high dose group.

Both Herth et al. [11] and Kramer et al. [9] observed that treatment related COPD exacerbations were found in eight of 25 patients (32%) and three of 20 patients (15%) respectively.

## Conclusions

- (Bio LVR) by using locally prepared fibrin glue could be an efficient method in treating advanced homogenous emphysema.
- Regarding safety and cost- effectiveness, the biological agent used was safe and low cost in comparison with synthetic agents.

### Limitations of the study

- Inability to perform multicenter study.
- Lack of comparison between the effect of BLVR in homogenous versus heterogenous emphysema.

### Recommendations

- Biological agents’ long-term efficacy in reducing lung volume could be assessed through additional clinical trials.
- Under investigation should be the combination of biological agents and other BioLVR techniques utilized in the same procedure.
- It is recommended that future investigations compare the effectiveness of bronchoscopic Bio LVR in cases of upper lobe predominant emphysema versus lower lobe predominant emphysema.

## Data Availability

The datasets generated and/or analyzed during the current study are not publicly available due to the belonging of data to the authors’ affiliated institute (Faculty of Medicine -Tanta University ), as well as to assure the privacy of respondents’ information. But data would be available from the corresponding author on reasonable request.
